# Dating violence in Portugal: how can it be handled in secondary schools and universities?

**DOI:** 10.3389/fgwh.2024.1456595

**Published:** 2024-09-19

**Authors:** Sofia Neves, Ariana Correia

**Affiliations:** ^1^ Department of Social and Behavioral Sciences, University of Maia, Maia, Portugal; ^2^ Interdisciplinary Centre for Gender Studies, University of Lisbon, Lisbon, Portugal

**Keywords:** dating, violence, Portugal, secondary schools, universities

## 1 Introduction

Dating violence is a pervasive and complex issue among youths who have intimate partner relationships (1). Studies on prevalence have shown that a significant number of school-aged young people are affected by physical, sexual, social, economic, or psychological violence in intimate partner relationships (2), with serious implications on health and social functioning (3).

In Portugal, this issue has garnered increasing attention from policymakers, educators, and researchers since the late 20th century because of broader social and legal changes and an increased awareness of domestic violence (4). Since the 1990s, key legislative and policy milestones have been achieved to ensure victims’ rights, in alignment with compromises adopted in the scope of international instruments, such as The Convention on Preventing and Combating Violence against Women and Domestic Violence, ratified in 2014.

In 2013, article 152 of the Portuguese Penal Code was amended, expanding the definition of domestic violence to include dating relationships in addition to marital, family, and similar relationships. This amendment was crucial, as it recognizes violence among youths in intimate partner relationships as a relevant problem. The current legal framework provides protection for victims of dating violence, criminalizes perpetrators, and mandates preventive measures.

In addition, the national plan “Portugal + Equal” (launched in 2018 by the Portuguese Government) includes addressing dating violence as a component of a comprehensive initiative aimed at promoting gender equality and combating all manifestations of gender-based violence and discrimination. The highlighted strategies include implementing school awareness programs to educate students about dating violence, developing primary preventive programs that target all levels of education, and organizing awareness-raising activities as part of the School Safety Program.^1^ The purpose of these acts is to instruct school-aged youths about the harmful consequences of dating violence and encourage a society that rejects violence and embraces gender equality (5).

Although national scientific studies concluded that the rates of victimization and perpetration of dating violence are similar for both genders (6), female victims tend to be more victimized and killed than male victims (7, 8). Moreover, typologies, dynamics, and consequences of dating violence are different according to gender, with women, for example, perpetrating less sexual violence and men re-offending more than women (9).

## 2 Data on dating violence in Portugal

Despite specific national official data on dating violence still being scarce, making it difficult to analyze its criminal trend, the more recent Annual Report of National Security (10) reveals that, in 2023, 30,461 cases of domestic violence were reported to police authorities. Of those, 85.5% were perpetrated in intimate relationships, with 4.9% referring to dating violence. Almost 70% of all victims of domestic violence were women.

The nationwide study on dating violence, encompassing more than 6,000 students from different educational levels (excluding university students), reveals substantial rates of acceptance and experience of dating violence in youth relationships, with manipulation, control, jealousy, and aggression being prevalent concerns (11). From the total number of young participants in the study who indicated having had or currently being in a dating relationship (*n* = 3,932), 63% (*n* = 2,477) reported experiencing at least one episode of violence. Male students legitimate a higher percentage of all forms of dating violence than female students.

In addition, the national study on dating violence in universities, with a sample of almost 5,000 university students, has been showing expressive outcomes regarding not only the prevalence of violence but also the relationship between practices and beliefs (12). Thus, the study emphasizes the extent to which dating violence is widespread, indicating that 53.7% (2,524) of students had suffered an episode of dating violence and 34% (1,599) had perpetrated one at least once during their lives. Moreover, a significant association between practices and beliefs was found. Hence, male students who conform to conventional gender norms accept more dating violence practices. Female students, on the other hand, typically have more egalitarian views on gender norms, with lower levels of legitimation of dating violence.

Considering data from official entities, non-governmental associations, and scientific studies, some evidence must be underlined. First, rates of domestic violence, particularly dating violence, are noteworthy, suggesting that women are more vulnerable to being victimized in intimate partner relationships than men. Even though gender differences are more evident in adult violent relationships, it is important to address the question “Why do female school-age youth seem to be as violent as males in dating relationships?”. Although violence occurs due to victims’ and perpetrators’ biological, psychological, cultural, economic, and political factors (13), some authors have been arguing that misinterpreting the notion of gender equality might be one of the explanations for mutual violence in youth relationships (14). As female empowerment has been gaining attention in recent years, young women become more independent, empowered, and aware of their rights, which may increase the likelihood of using violent conduct to protect themselves. The figure of self-defense is not new in the literature, with Hamby (15), for example, defending that women's physical violence in intimate relationships should be understood as female resistance to male violence.

Second, even though women perpetrate psychological, physical, social, and economic violence toward men, they are rarely sexually abusive, and hardly ever re-offend. When men hold traditional beliefs about gender roles, sexual violence toward women is often an expression of power and control (16). In the same sense, low rates of sexual violence against men can be explained by their shame in complaining to police authorities, as the perpetrators are women or other men. Moreover, women are at a greater risk of being murdered, which means that besides analyzing dating violence prevalence, it is necessary to comprehend its typologies, motives, dynamics, and consequences, using a gender-based and intersectional approach (17).

Third, although Portugal has been investing in legal instruments, national strategies, and measures, the rates of dating violence among secondary and university students are still high, suggesting that the prevention initiatives are not providing effective results. The dating violence prevention programs implemented in Portuguese schools are generally perceived as effective, although their impacts concerning violence reduction are limited (18).

## 3 Conclusion

As most cases of dating violence occur in secondary schools and universities, specific programs, specialized services, and inter-agency coordination need to be developed to prevent and combat it. Thus, each school or university must have an internal policy on dating violence, complete with a regulation and an action flowchart. As per the internal policy, an analysis of the social and cultural environment must be conducted, aiming to establish a comprehensive map of dating violence through surveys and interviews but also to increase awareness among young people. Sharing data on the typologies, dynamics, and consequences of dating violence might result in a higher alertness among victims and mobilization among bystanders, as well as decreased violence (19). The programs should take a gender and intersectional-based approach and be multilevel, involving all the school or university community, teaching staff, non-teaching staff, students, and administration. In addition, a multimethod approach should be applied, using collaborative and participatory techniques.

Victim support services, including hotlines and counseling, must be created to offer immediate and specialized assistance and long-term support. The consequences of dating violence are severe regarding victims’ mental health. Anxiety and depression symptomatology, along with substance abuse, self-injury behaviors, and suicidal ideation, are common effects constraining life quality and wellbeing (20). Providing resources to enhance victims’ skills to face victimization experiences is essential for empowering them and promoting their resilience in the aftermath of trauma. Secondary schools and universities may contribute to improving youth mental health. In addition, services should be provided to offenders to reduce the rates of dating violence and recidivism. Allocating more resources to education and support services should be a priority of schools and universities.

Inter-agency coordination requires better and deeper relationships among various agencies and stakeholders who have responsibilities in addressing dating violence. Fostering a culture free of violence, in which all parts contribute to forming safe communities, can have a greater impact on social and interpersonal dynamics.

In addition, it is necessary to assess the internal policy's effectiveness by examining various indicators, such as the availability and accessibility of support services, public awareness levels, and reported incidents of dating violence.

Portugal has made significant strides in addressing dating violence. However, continued efforts are needed to overcome existing challenges and enhance the effectiveness of its prevention and methods to combat it. By focusing on education, support services, and inter-agency coordination, the country can further strengthen its response to dating violence and ensure healthy relationships for its youth.

## Footnotes

^1^The Safe School Program is a collaborative effort between the Ministry of Internal Administration and the Ministry of Education in Portugal. Its primary objective is to guarantee the security of school premises and their surrounding areas. This nationwide initiative encompasses all non-higher education institutions, encompassing both public and commercial entities. The main objective is to mitigate hazardous behaviors and minimize instances of insecurity in schools by engaging teams from the Public Security Police (PSP) and the National Republican Guard (GNR), with the assistance of security personnel from the Ministry of Education.
