# Corrigendum: Dating violence in Portugal: how can it be handled in secondary schools and universities?

**DOI:** 10.3389/fgwh.2025.1576105

**Published:** 2025-03-11

**Authors:** Sofia Neves, Ariana Correia

**Affiliations:** ^1^ Department of Social and Behavioral Sciences, University of Maia, Maia, Portugal; ^2^ Interdisciplinary Centre for Gender Studies, University of Lisbon, Lisbon, Portugal

**Keywords:** dating, violence, Portugal, secondary schools, universities

A Corrigendum on Dating violence in Portugal: how can it be handled in Secondary Schools and Universities? By Neves S and Correia A (2024). Front. Glob. Womens Health 5. doi: 10.3389/fgwh.2024.1456595

Corrigendum on: Neves S and Correia A (2024) Dating violence in Portugal: how can it be handled in secondary schools and universities?. Front. Glob. Womens Health 5:1456595. doi: 10.3389/fgwh.2024.1456595

In the published article, there was an error in the data quoted from reference 10.

A correction has been made to section 2 Data on dating violence in Portugal, paragraph 1. This sentence previously stated:

“Despite specific national official data on dating violence still being scarce, making it difficult to analyze its criminal trend, the more recent Report of National Security (10) reveals that among the 40,361 reports of domestic violence to police authorities, 4.9% (*n* = 1,681) refer to dating violence. Almost 70% of all victims of domestic violence were women”.

The corrected sentence appears below:

“Despite specific national official data on dating violence still being scarce, making it difficult to analyze its criminal trend, the more recent Annual Report of National Security (10) reveals that, in 2023, 30,461 cases of domestic violence were reported to police authorities. Of those, 85.5% were perpetrated in intimate relationships, with 4.9% referring to dating violence. Almost 70% of all victims of domestic violence were women”.

The authors apologize for this error and state that this does not change the scientific conclusions of the article in any way. The original article has been updated.

